# Mental distress and incident functional disability among a rural ageing population in South Africa

**DOI:** 10.1002/gps.5840

**Published:** 2022-11-12

**Authors:** Supa Pengpid, Karl Peltzer

**Affiliations:** ^1^ Department of Health Education and Behavioral Sciences Faculty of Public Health Mahidol University Bangkok Thailand; ^2^ Department of Public Health Sefako Makgatho Health Sciences University Pretoria South Africa; ^3^ Department of Healthcare Administration College of Medical and Health Science Asia University Taichung Taiwan; ^4^ Department of Psychology University of the Free State Bloemfontein South Africa; ^5^ Department of Psychology College of Medical and Health Science Asia University Taichung Taiwan

**Keywords:** functional disability, longitudinal study, mental symptoms, South Africa

## Abstract

**Objectives:**

The aim of the study was to investigate the association between mental symptoms and incident functional disability among middle‐age and older adults in South Africa.

**Methods:**

Longitudinal data from two consecutive population surveys (2014/2015–2018/2019) in Agincourt, South Africa, were analysed.

**Results:**

In total, 298 of 3813 participants without functional disability in wave 1 (8.8%) had functional disability in wave 2. The prevalence of baseline functional disability was 9.1%.

In the fully adjusted models for people without functional disability at baseline, depressive symptoms (AOR: 1.74, 95% CI: 1.08–2.80) among men and lower life satisfaction among men (AOR: 0.86, 95% CI: 0.80–0.93) and among women (AOR: 0.90, 95% CI: 0.83–0.98) increased the odds of incident functional disability. Posttraumatic stress disorder symptoms, poor sleep quality, restless sleep, and loneliness were not significantly associated with incident functional disability.

**Conclusions:**

Depressive symptoms among men and lower life satisfaction among both sexes were independently associated with incident functional disability in ageing rural South Africans.

## Key points

- Among middle‐aged and older adults in South Africa without functional disability at baseline, depressive symptoms among men and lower life satisfaction among both sexes increased the odds of incident functional disability.
- Posttraumatic stress disorder symptoms, poor sleep quality, restless sleep, and loneliness were not significantly associated with incident functional disability.

## 1 INTRODUCTION

Disabilities in activities of daily living (ADLs) are a major public health problem in the ageing population.^1^ The prevalence of functional ADL disability in middle‐aged adults (55–65 years) from 23 countries was 7.9% among women and 6.9% among men.^2^ Among middle‐aged and older adults in six middle‐income countries, the prevalence of ADL disability was the highest in India (55.7%) and lowest in China (16.2%).^3^ Among older adults in South Africa, 37.2% of the participants had “moderate or severe and/or very severe functional disability.”^4^

Functional disability is associated with increased hospitalization,^5^ poor self‐rated quality of life,^6^ and mortality.^7^, ^8^, ^9^ Therefore, it would be important to identify modifiable factors, such as mental symptoms, that can delay or prevent functional disability from occurring in older age.^10^ In an earlier review, depression and anxiety in old age were found to be a risk factor for disability.^11^ Several recent studies in high‐income countries among older adults (psychological distress in Japan,^12^ severe depressive symptoms in Japan,^13^ and depressive symptoms among Chinese Elderly in Chicago^14^), and in middle‐income countries and (depressive symptoms in Brazil,^15^ and depressive symptoms in China^16^) all showed an association with incident ADL disability. In a longitudinal study in the UK (mean age 69 years), loneliness was associated with incident ADL disability,^17^ while in a study in China (≥50 years), social isolation but not loneliness was associated with incident ADL disability among women.^10^ In the USA, among people aged 24–75 years, “chronic sleep problems predicted a higher risk of onset and increases in functional limitations 9–10 years later,”^18^ non‐disabled older adults with more sleep complaints had higher odds of developing ADL disability in the USA,^19^ while among older adults in Japan, long sleep duration and excessive sleepiness during the day were associated incident disability.^20^ In China, high life satisfaction was “independently associated with longer disability‐free survival older adults.”^21^

In cross‐sectional studies in Africa, higher depression scores and major depressive disorder were associated with functional disability among older adults in Nigeria,^22^, ^23^ and among middle‐aged and older adults in Ghana, loneliness was associated with physical functioning impairment,^24^ and among middle‐aged and older South Africans, lower quality of life was associated with functional disability.^4^ None of the longitudinal studies reviewed were conducted in African countries, and it is unclear to what extent the associations of mental symptoms with functional disability apply to African populations. Therefore, this investigation aimed to evaluate the association between mental symptoms and incident ADL disability in a longitudinal study in rural South Africa.

## 2 METHODS

### 2.1 Participants and procedures

Longitudinal data from two consecutive waves (2014/2015–2018/2019) of the “Health and Ageing in Africa: A Longitudinal Study of an INDEPTH Community in South Africa (HAALSI)” were analysed. Full details of the sampling methodology has been published.^25^ Wave 1 included 5059 persons (≥40 years) (response rate 85.9%),^25^ and in wave 24,176 people were followed up (12% had died, 5% declined participation, and <1% could not be traced; response rate 94%). Measurements were administered by trained field workers in the homes of participants using computer‐assisted personal interviewing (CAPI).^25^ The study was approved by the “University of the Witwatersrand Human Research Ethics Committee (ref. M141159), the Harvard T.H. Chan School of Public Health, Office of Human Research Administration (ref. C13–1608–02), and the Mpumalanga Provincial Research and Ethics Committee”.^25^ Participants provided their written informed consent.

## 3 MEASURES

### 3.1 Outcome variable

In waves 1 and 2, functional disability was assessed with six yes‐no items (“bathing, dressing, eating, getting in/out of bed using the toilet and walking”) on the ADL scale,^26^ Cronbach's alpha was 0.86 at baseline and 0.88 at follow‐up in this study.

### 3.2 Exposure variables

*Depressive symptoms* (scores ≥3) was classified with the “Centre for Epidemiological Studies‐Depression Scale eight‐item scale (CES‐D 8).”^27^, ^28^ (Cronbach's alpha was 0.66 in the first survey).

*Posttraumatic Stress Disorder* (PTSD) was defined as ≥4 PTSD symptoms.^29^ (Cronbach's alpha 0.83).

*Poor sleep quality* (≥5 scores) was obtained from the “Brief Version of the Pittsburgh Sleep Quality Index (B‐PSQI),” which included five sections: “self‐reported sleep quality, sleep latency, sleep duration, habitual sleep efficiency and sleep disturbances” during the past month.^30^

*Restless sleep* as sourced from the item, “Much of the time in the past week, your sleep was restless”, “Yes”.^27^

*Loneliness* was defined as “Much of the time in the past week, you felt lonely.” (Yes).^27^

*Life satisfaction* was derived from the item, “All things considered, how satisfied are you with your life as a whole these days? Use a 0 to 10 scale, where 0 is dissatisfied and 10 is satisfied.”^25^

#### 3.2.1 Covariates

*Social and demographic data* included education, age, sex, marital, and household wealth status.^25^

*Current tobacco use* was defined as current smokeless tobacco use and/or current smoking.^25^

*Alcohol dependence* was assessed with the 4‐item Cut, Annoyed, Guilty and Eye scale.^31^ (Cronbach's alpha was 0.82).

*The levels of physical activity* were evaluated using the “General Physical Activity Questionnaire (GPAQ)”.^32^, ^33^

*Sedentary behaviour* included one item “time usually spend sitting or reclining on a typical day?” from the GPAQ,^32^ and grouped into “<4 h, 4 to <8 h and eight or more hours per day”.^34^

*Body Mass Index* (BMI) was measured and classified using World Health Organization criteria.^35^

*Hypertension* was measured and defined based on National Committee criteria.^36^

*Dyslipidemia* was defined as: “total cholesterol >6.21 mmol/L, HDL‐C < 1.19 mmol/L, LDL‐C > 4.1 mmol/L, triglycerides >2.25 mmol/L, or ever diagnosed or medication use for high cholesterol”.^25^

*Diabetes* was “classified with fasting glucose (defined as > 8 h) > 7 mmol/L (126 mg/dl), ever diagnosed or medication use for diabetes.”^25^

### 3.3 Data analysis

Frequency distribution of participants with incident functional disability is described. In the first longitudinal logistic regression model people with functional disability at baseline were excluded, leaving a sample of 4573 individuals to estimate incident functional disability. Mental symptoms were the main predictors and the outcome variable included incident functional disability. Logistic regression models included four steps: unadjusted model, model 1: adjusted for sociodemographic variables (education, sex, age, marital and wealth status); model II: Adjusted for Model 1 variables plus behavioural variables (alcohol use, sedentary behaviour, physical activity and BMI); and model III: adjusted for previous variables, plus chronic conditions (hypertension, dyslipidemia, and diabetes). *p* < 0.05 was considered statistically significant. Inverse probability weights accounting for mortality and attrition at follow‐up were applied to longitudinal data analyses. All analyzes were conducted using StataSE 15.0 (College Station, TX, USA).

## 4 RESULTS

### 4.1 Sample characteristics by incident functional disability

In total, 298 of 3813 participants without functional disability in wave 1 (8.8%) had functional disability in wave 2. The prevalence of baseline functional disability was 9.1%, and incident functional disability as 8.9% in men and 8.7% in women. Further details (see Table 1).

**TABLE 1:** Sample characteristics by incident functional disability, Agincourt, South Africa, 2014–2019

			Incident functional disability	
		Sample	Men	Women
Baseline variables		*N* (%)	%	%
Age (in years)	40–49	884 (17.6)	1.6	1.6
	50–59	1358 (27.1)	5.4	2.8
	60–69	1274 (25.4)	5.8	7.4
	70–79	918 (18.3)	14.2	15.8
	≥80	583 (11.6)	31.2	30.2
Sex	Female	2713 (53.6)		
	Male	2346 (46.4)		
Education	None	2307 (49.1)	12.3	11.8
	1–7 years	1613 (32.0)	8.5	8.8
	8–11	537 (10.7)	4.9	1.1
	12 or more	585 (11.6)	3.8	1.9
Marital status	Married/cohabiting	2575 (50.9)	7.6	4.1
	Not married	2480 (49.1)	11.9	11.4
Wealth index	Low	2047 (40.5)	11.6	9.5
	Middle	991 (19.6)	8.0	8.3
	High	2021 (39.9)	6.9	8.3
Current tobacco use	No	4264 (84.4)	9.5	7.9
	Yes	790 (15.6)	7.3	17.1
Alcohol dependence	No	4988 (98.7)	9.2	8.8
	Yes	68 (1.3)	1.6	0.0
Physical activity	Low	221 (44.0)	12.5	13.6
	Moderate	1143 (22.7)	6.8	4.7
	High	1674 (33.3)	6.8	5.7
Sedentary behaviour	Low	2675 (55.9)	6.7	6.3
	Moderate	1632 (34.1)	10.6	10.8
	High	475 (9.9)	14.6	15.0
Body mass index	Normal	1719 (36.7)	7.1	8.5
	Under	258 (5.5)	13.9	11.1
	Overweight	1328 (28.3)	10.5	8.1
	Obesity	1384 (29.5)	8.0	8.0
Hypertension	No	2052 (41.6)	5.5	5.5
	Yes	2884 (58.4)	11.8	10.8
Diabetes	No	4093 (88.0)	8.5	8.2
	Yes	559 (12.0)	18.3	13.5
Dyslipidaemia	No	2389 (56.2)	8.7	8.5
	Yes	1862 (43.8)	11.0	8.6
Depressive symptoms	No	4092 (83.0)	7.5	7.4
	Yes	837 (17.0)	14.9	12.9
PTSD symptoms	No	4697 (96.2)	8.7	8.3
	Yes	238 (4.8)	7.3	7.0
Poor sleep quality	No	3904 (83.0)	8.5	7.8
	Yes	801 (17.0)	10.3	11.8
Restless sleep	No	3320 (67.1)	8.1	6.5
	Yes	1626 (32.9)	9.8	11.7
Loneliness	No	4280 (86.5)	8.0	7.3
	Yes	669 (13.5)	12.9	14.7

		M (SD)	M (SD)	M (SD)
Life satisfaction	Scale (0–10)	6.7 (2.4)	6.2 (2.4)	6.0 (2.4)

### 4.2 Associations between mental symptoms and incident functional disability among men

In the fully adjusted models for people without functional disability at baseline, depressive symptoms (AOR: 1.74, 95% CI: 1.08–2.80) and lower life satisfaction (AOR: 0.86, 95% CI: 0.80–0.93) increased the odds of incident functional disability among men. Posttraumatic Stress Disorder symptoms, poor sleep quality, restless sleep and loneliness were not significantly associated with incident functional disability (see Table 2).

**TABLE 2:** Longitudinal association between mental symptoms and incident functional disability among men, Agincourt, South Africa, 2014–2019

Baseline variable	Unadjusted model	Model 1	Model 2	Model 3
	Odds ratio (95% CI)	Odds ratio (95% CI)	Odds ratio (95% CI)	Odds ratio (95% CI)
Depressive symptoms				
No	1 (Reference)	1 (Reference)	1 (Reference)	1 (Reference)
Yes	2.12 (1.47, 3.06)***	1.76 (1.18, 2.63)**	1.71 (1.11, 2.61)*	1.74 (1.08, 2.80)*
PTSD symptoms				
No	1 (Reference)	1 (Reference)	1 (Reference)	1 (Reference)
Yes	0.91 (0.39, 2.14)	0.75 (0.30, 1.86)	0.81 (0.33,2.03)	0.92 (0.36, 2.37)
Poor sleep quality				
No	1 (Reference)	1 (Reference)	1 (Reference)	1 (Reference)
Yes	1.27 (0.81, 1.89)	1.19 (0.79, 1.82)	1.23 (0.80, 1.90)	1.32 (0.98, 1.79)
Restless sleep				
No	1 (Reference)	1 (Reference)	1 (Reference)	1 (Reference)
Yes	1.25 (0.89, 1.74)	1.03 (0.72,1.47)	1.06 (0.73, 1.54)	1.02 (0.67, 1.53)
Loneliness				
No	1 (Reference)	1 (Reference)	1 (Reference)	1 (Reference)
Yes	1.53 (1.01, 2.32)*	1.00 (0.63, 1,59)	0.88 (0.52, 1.49)	0.91 (0.52, 1.60)
Life satisfaction				
Scale	0.89 (0.83, 0.94)***	0.88 (0.83, 0.95)**	0.87 (0.81, 0.94)***	0.86 (0.80, 0.93)***

*Note*: Model 1: Adjusted for sociodemographic variables. Model II: Adjusted for Model 1 variables, plus behavioural variables. Model III: Adjusted for Model I and II variables, plus chronic conditions.

*p < 0.05; **p < 0.01; ***p < 0.001.

### 4.3 Associations between mental symptoms and incident functional disability among women

Table 3 shows, based on the longitudinal analysis, the associations between mental symptoms and incident functional disability among women. In fully adjusted models, lower life satisfaction (AOR: 0.90, 95% CI: 0.83–0.98) was significantly associated with incident functional disability. Depressive symptoms, poor sleep quality, restless sleep, and loneliness were only found in the unadjusted models significantly associated with incident functional disability (see Table 3).

**TABLE 3:** Longitudinal association between mental symptoms and incident functional disability among women, Agincourt, South Africa, 2014–2019

Baseline variable	Unadjusted model	Model 1	Model 2	Model 3
	Odds ratio (95% CI)	Odds ratio (95% CI)	Odds ratio (95% CI)	Odds ratio (95% CI)
Depressive symptoms				
No	1 (Reference)	1 (Reference)	1 (Reference)	1 (Reference)
Yes	1.82 (1.30, 2.56)***	1.30 (0.90, 1.88)	1.24 (0.84, 1.83)	1.01 (0.63, 1.63)
PTSD symptoms				
No	1 (Reference)	1 (Reference)	1 (Reference)	1 (Reference)
Yes	0.80 (0.40, 1.61)	0.63 (0.29,1.38)	0.60 (0.26, 2.40)	0.87 (0.37, 2.05)
Poor sleep quality				
No	1 (Reference)	1 (Reference)	1 (Reference)	1 (Reference)
Yes	1.60 (1.11, 2.28)*	1.43 (0.97, 2.11)	1.26 (0.82, 1.93)	1.57 (0.96, 2.54)
Restless sleep				
No	1 (Reference)	1 (Reference)	1 (Reference)	1 (Reference)
Yes	1.91 (1.43, 2.56)***	1.28 (0.93, 1.79)	1.24 (0.89, 1.73)	1.17 (0.80, 1.71)
Loneliness				
No	1 (Reference)	1 (Reference)	1 (Reference)	1 (Reference)
Yes	2.20 (1.55, 3.11)***	1.45 (0.99, 2.01)	1.44 (0.93, 2.23)	1.31 (0.81, 2.12)
Life satisfaction				
Scale	0.87 (0.82, 0.92)***	0.90 (0.84, 0.96)***	0.90 (0.84, 0.97)**	0.90 (0.83, 0.98)*

*Note*: Model 1: Adjusted for sociodemographic variables. Model II: Adjusted for Model 1 variables, plus behavioural variables. Model III: Adjusted for Model I and II variables, plus chronic conditions.

*p < 0.05; **p < 0.01; ***p < 0.001.

## 5 DISCUSSION

In this first prospective investigation on mental distress and functional disability among middle‐aged and older adults in Africa, we found that higher depressive symptoms at baseline among men and lower life satisfaction among both sexes increased the odds of incident functional disability after 4 years. Poor sleep quality, PTSD symptoms, restless sleep and loneliness were not significantly associated with incident functional disability. The findings appear to confirm previous results^11^, ^12^, ^13^, ^14^, ^15^, ^21^ of an association between depressive symptoms, lower life satisfaction, and incident functional disability, extending previous knowledge on the relationship between depressive symptoms, lower life satisfaction, and disability onset in an African ageing population. Several mechanisms for the development of functional disability from depressive symptoms include the continuous presence of somatic depressive symptoms, poorer management of chronic medical conditions, and increased health risk behaviours, all of which can contribute directly or indirectly to the decline in ADL disability.^14^, ^37^, ^38^

However, some previous research^11^, ^17^, ^18^, ^19^ showed an association between sleep disturbance, anxiety, loneliness, and incident functional disability, while we did not find any significant association in adjusted analysis. However, among women, in the unadjusted analysis, poor sleep quality, restless sleep, and loneliness were positively associated with incident functional disability. Loneliness was only assessed with a single item, which may have prevented us from detecting significant effects on ADL disability, and future studies may include a full loneliness scale.

### 5.1 Study limitations

Some data, including depressive symptoms, sleep parameters, and disability, were assessed by self‐report and not verified by clinical evaluation and actigraphy or polysomnography, which may have led to recall bias. Additionally, individuals without ADL disability in wave 1 may have had ADL disability before. In wave two only ADL and not Instrumental (I)ADL was assessed, which hindered us to calculate estimates of mental symptoms on instrumental activities of daily living disability. We were not able to determine the degree or severity of functional limitation, since a Yes‐No response format was used. However, activity limitations were dichotomized into at least one limitation and no limitation following previous studies.^10^, ^16^ Furthermore, the study power was limited due to the small number of incident functional disability cases.

## 6 CONCLUSIONS

Depressive symptoms among men and lower life satisfaction among both sexes were independently associated with incident functional disability in ageing rural South Africans. Enhanced screening and treatment of depressive symptoms and low life satisfaction among men and/or women may reduce functional disability in this population.

## AUTHOR CONTRIBUTION

“All authors fulfil the criteria for authorship. SP and KP conceived and designed the research, performed statistical analysis, drafted the manuscript, and made critical revisions of the manuscript for key intellectual content. All authors read and approved the final version of the manuscript and have agreed to the authorship and order of authorship for this manuscript.”

## CONFLICT OF INTEREST

The authors declare no conflict of interest.

## ETHICS STATEMENT

The study was approved by the “University of the Witwatersrand Human Research Ethics Committee (ref. M141159), the Harvard T.H. Chan School of Public Health, Office of Human Research Administration (ref. C13–1608–02), and the Mpumalanga Provincial Research and Ethics Committee”.^6^ All participants provided written informed consent.

## Data Availability

The data that support the findings of this study are openly available in Harvard Centre for Population and Development Studies (HCPDS at https://dataverse.harvard.edu/.

## References

[1] Maresova P , Javanmardi E , Barakovic S , et al. Consequences of chronic diseases and other limitations associated with old age ‐ a scoping review. BMC Publ Health. 2019;19(1):1431. 10.1186/s12889-019-7762-5 PMC6823935 31675997

[2] Wang S , Phillips D , Lee J . Disability prevalence in midlife (aged 55‐65 years): cross‐Country comparisons of gender differences and time trends. Womens Midlife Health. 2021;7(1):1. 10.1186/s40695-020-00061-0 33386080 PMC7777402

[3] Lestari SK , Ng N , Kowal P , Santosa A . Diversity in the factors associated with ADL‐related disability among older people in six middle‐income countries: a cross‐country comparison. Int J Environ Res Publ Health. 2019;16(8):1341. 10.3390/ijerph16081341 PMC6518276 31013975

[4] Phaswana‐Mafuya N , Peltzer K , Ramlagan S , Chirinda W , Kose Z . Social and health determinants of gender differences in disability amongst older adults in South Africa. Health SA Gesondheid. 2013;18(1):9. Art. #728. 10.4102/hsag.v18i1.728

[5] Chen C , Lim JT , Chia NC , et al. The long‐term impact of functional disability on hospitalization spending in Singapore. J Econ Ageing. 2019;14:100193. 10.1016/j.jeoa.2019.02.002 31857943 PMC6922027

[6] Yau PN , Foo CJ , Cheah NL , Tang KF , Lee SW . The prevalence of functional disability and its impact on older adults in ASEAN region: a systematic review and meta‐analysis. Epidemiol Health. 2022:e2022058. 10.4178/epih.e2022058 35843601 PMC9754909

[7] Forman‐Hoffman VL , Ault KL , Anderson WL , et al. Disability status, mortality, and leading causes of death in the United States community population. Med Care. 2015;53(4):346‐354. 10.1097/MLR.0000000000000321 25719432 PMC5302214

[8] Langballe EM , Tangen GG , Engdahl B , Strand BH . Increased mortality risk for adults aged 25‐44 years with long‐term disability: a prospective cohort study with a 35‐year follow‐up of 30, 080 individuals from 1984‐2019 in the population‐based HUNT study. Lancet Reg Health Eur. 2022;22:100482. 10.1016/j.lanepe.2022.100482 36039147 PMC9418547

[9] Berthé A , Berthé‐Sanou L , Somda S , et al. The key actors maintaining elders in functional autonomy in Bobo‐Dioulasso (Burkina Faso). BMC Publ Health. 2014;14(1):689. 10.1186/1471-2458-14-689 PMC4094536 24997509

[10] Guo L , An L , Luo F , Yu B . Social isolation, loneliness and functional disability in Chinese older women and men: a longitudinal study. Age Ageing. 2021;50(4):1222‐1228. 10.1093/ageing/afaa271 33352582

[11] Lenze EJ , Rogers JC , Martire LM , et al. The association of late‐life depression and anxiety with physical disability: a review of the literature and prospectus for future research. Am J Geriatr Psychiatr. 2001;9(2):113‐135. 10.1097/00019442-200105000-00004 11316616

[12] Tomata Y , Watanabe T , Tanji F , Zhang S , Sugawara Y , Tsuji I . The impact of psychological distress on incident functional disability in elderly Japanese: the Ohsaki cohort 2006 study. Int J Environ Res Publ Health. 2018;15(11):2502. 10.3390/ijerph15112502 PMC6265961 30413102

[13] Kondo N , Kazama M , Suzuki K , Yamagata Z . Impact of mental health on daily living activities of Japanese elderly. Prev Med. 2008;46(5):457‐462. 10.1016/j.ypmed.2007.12.007 18258290

[14] Kong D , Solomon P , Dong X . Depressive symptoms and onset of functional disability over 2 Years: a prospective cohort study. J Am Geriatr Soc. 2019;67(S3):S538‐S544. 10.1111/jgs.15801 31403199 PMC9942515

[15] Torres JL , Castro‐Costa E , Mambrini JVM , et al. Depressive symptoms, emotional support and activities of daily living disability onset: 15‐year follow‐up of the Bambuí (Brazil) Cohort Study of Aging. Cad Saúde Pública. 2018;34(7):e00141917. 10.1590/0102-311X00141917 30088584

[16] Peng S , Wang S , Feng XL . Multimorbidity, depressive symptoms and disability in activities of daily living amongst middle‐aged and older Chinese: evidence from the China Health and Retirement Longitudinal Study. J Affect Disord. 2021;295:703‐710. 10.1016/j.jad.2021.08.072 34517243

[17] Shankar A , McMunn A , Demakakos P , Hamer M , Steptoe A . Social isolation and loneliness: prospective associations with functional status in older adults. Health Psychol. 2017;36(2):179‐187. 10.1037/hea0000437 27786518

[18] Friedman EM . Self‐reported sleep problems prospectively increase risk of disability: findings from the survey of midlife development in the United States. J Am Geriatr Soc. 2016;64(11):2235‐2241. 10.1111/jgs.14347 27626617 PMC5118074

[19] Park M , Buchman AS , Lim AS , Leurgans SE , Bennett DA . Sleep complaints and incident disability in a community‐based cohort study of older persons. Am J Geriatr Psychiatr. 2014;22(7):718‐726. 10.1016/j.jagp.2012.12.023 PMC3735667 23567404

[20] Nakakubo S , Doi T , Makizako H , et al. Sleep duration and excessive daytime sleepiness are associated with incidence of disability in community‐dwelling older adults. J Am Med Dir Assoc. 2016;17(8):768.e1‐5. 10.1016/j.jamda.2016.05.020 27397602

[21] Wu W , Shang Y , Calderón‐Larrañaga A , et al. Association of life satisfaction with disability‐free survival: role of chronic diseases and healthy lifestyle. Age Ageing. 2021;50(5):1657‐1665. 10.1093/ageing/afab086 34120170

[22] Gureje O , Kola L , Afolabi E , Olley BO . Determinants of quality of life of elderly Nigerians: results from the Ibadan study of ageing. Afr J Med Med Sci. 2008;37(3):239‐247. 18982816 PMC2820711

[23] Akosile CO , Mgbeojedo UG , Maruf FA , Okoye EC , Umeonwuka IC , Ogunniyi A . Depression, functional disability and quality of life among Nigerian older adults: prevalences and relationships. Arch Gerontol Geriatr. 2018;74:39‐43. 10.1016/j.archger.2017.08.011 28954240

[24] Gyasi RM , Peprah P , Abass K , et al. Loneliness and physical function impairment: perceived health status as an effect modifier in community‐dwelling older adults in Ghana. Prev Med Rep. 2022;26:101721. 10.1016/j.pmedr.2022.101721 35141124 PMC8814641

[25] Gómez‐Olivé FX , Montana L , Wagner RG , et al. Cohort profile: health and ageing in Africa: a longitudinal study of an INDEPTH community in South Africa (HAALSI). Int J Epidemiol. 2018;47(3):689‐690j. 10.1093/ije/dyx247 29325152 PMC6005147

[26] Katz S , Ford AB , Heiple KG , Newill VA . Studies of illness in the aged: recovery after fracture of the hip. J Gerontol. 1964;19(3):285‐293. 10.1093/geronj/19.3.285 14179683

[27] Radloff LS . The CES‐D scale: a self‐report depression scale for research in the general population. Appl Psychol Meas. 1977;1(3):385‐401. 10.1177/014662167700100306

[28] Steffick DE . Documentation of Affective Functioning Measures in the Health and Retirement Study. Survey Research Center University of Michigan Ann Arbor, MI; 2000. Accessed November 25, 2021. URL: chrome‐extension. http://efaidnbmnnnibpcajpcglclefindmkaj/viewer.html?pdfurl=https%3A%2F%2Fhrs.isr.umich.edu%2Fsites%2Fdefault%2Ffiles%2Fbiblio%2Fdr‐005.pdf%26clen=3036979%26chunk=true

[29] Breslau N , Peterson EL , Kessler RC , Schultz LR . Short screening scale for DSM‐IV posttraumatic stress disorder. Am J Psychiatr. 1999;156(6):908‐911. 10.1176/ajp.156.6.908 10360131

[30] Sancho‐Domingo C , Carballo JL , Coloma‐Carmona A , Buysse DJ . Brief version of the Pittsburgh Sleep Quality Index (B‐PSQI) and measurement invariance across gender and age in a population‐based sample. Psychol Assess. 2021;33(2):111‐121. 10.1037/pas0000959 33119375

[31] Ewing JA . Detecting alcoholism. The CAGE questionnaire. JAMA. 1984;252(14):1905‐1907. 10.1001/jama.252.14.1905 6471323

[32] Armstrong T , Bull F . Development of the world health organization global physical activity questionnaire (GPAQ). J Public Health. 2006;14(2):66‐70. 10.1007/s10389-006-0024-x

[33] World Health Organization (WHO) . Global Physical Activity Surveillance; 2009. Accessed October 2, 2021. http://www.who.int/chp/steps/GPAQ/en/index.html

[34] van der Ploeg HP , Chey T , Korda RJ , Banks E , Bauman A . Sitting time and all‐cause mortality risk in 222 497 Australian adults. Arch Intern Med. 2012;172(6):494‐500. 10.1001/archinternmed.2011.2174 22450936

[35] World Health Organization (WHO) . Obesity: Preventing and Managing the Global Epidemic ‐ Report of a WHO Consultation. World Health Organization; 2000. 11234459

[36] Chobanian AV , Bakris GL , Black HR , et al. The seventh report of the joint national committee on prevention, detection, evaluation, and treatment of high blood pressure: the JNC 7 report. JAMA. 2003;289(19):2560‐2572. 10.1001/jama.289.19.2560 12748199

[37] Penninx BW , Leveille S , Ferrucci L , van Eijk JT , Guralnik JM . Exploring the effect of depression on physical disability: longitudinal evidence from the established populations for epidemiologic studies of the elderly. Am J Publ Health. 1999;89(9):1346‐1352. 10.2105/ajph.89.9.1346 PMC1508750 10474551

[38] Katon WJ . Epidemiology and treatment of depression in patients with chronic medical illness. Dialogues Clin Neurosci. 2011;13(1):7‐23. 10.31887/DCNS.2011.13.1/wkaton 21485743 PMC3181964

